# Comparing code-free deep learning models to expert-designed models for detecting retinal diseases from optical coherence tomography

**DOI:** 10.1186/s40942-024-00555-3

**Published:** 2024-04-26

**Authors:** Samir Touma, Badr Ait Hammou, Fares Antaki, Marie Carole Boucher, Renaud Duval

**Affiliations:** 1 Department of Ophthalmology, Université de Montréal, Montreal, Québec Canada; 2 Centre Universitaire d’Ophtalmologie (CUO), Hôpital Maisonneuve-Rosemont, CIUSSS de l’Est- de-l’Île-de-Montréal, 5415 boulevard de l’Assomption, H1T 2M4 Montreal, QC Canada; 3 Department of Ophthalmology, Centre Hospitalier de l’Université de Montréal (CHUM), Montreal, QC Canada; 4 The CHUM School of Artificial Intelligence in Healthcare (SAIH), Centre Hospitalier de l’Université de Montréal (CHUM), Montreal, QC Canada

**Keywords:** Optical coherence tomography, Artificial intelligence, Code-free machine learning

## Abstract

**Background:**

Code-free deep learning (CFDL) is a novel tool in artificial intelligence (AI). This study directly compared the discriminative performance of CFDL models designed by ophthalmologists without coding experience against bespoke models designed by AI experts in detecting retinal pathologies from optical coherence tomography (OCT) videos and fovea-centered images.

**Methods:**

Using the same internal dataset of 1,173 OCT macular videos and fovea-centered images, model development was performed simultaneously but independently by an ophthalmology resident (CFDL models) and a postdoctoral researcher with expertise in AI (bespoke models). We designed a multi-class model to categorize video and fovea-centered images into five labels: normal retina, macular hole, epiretinal membrane, wet age-related macular degeneration and diabetic macular edema. We qualitatively compared point estimates of the performance metrics of the CFDL and bespoke models.

**Results:**

For videos, the CFDL model demonstrated excellent discriminative performance, even outperforming the bespoke models for some metrics: area under the precision-recall curve was 0.984 (vs. 0.901), precision and sensitivity were both 94.1% (vs. 94.2%) and accuracy was 94.1% (vs. 96.7%). The fovea-centered CFDL model overall performed better than video-based model and was as accurate as the best bespoke model.

**Conclusion:**

This comparative study demonstrated that code-free models created by clinicians without coding expertise perform as accurately as expert-designed bespoke models at classifying various retinal pathologies from OCT videos and images. CFDL represents a step forward towards the democratization of AI in medicine, although its numerous limitations must be carefully addressed to ensure its effective application in healthcare.

**Supplementary Information:**

The online version contains supplementary material available at 10.1186/s40942-024-00555-3.

## Introduction

Over the past decade, artificial intelligence (AI) experienced great strides and various algorithms have been developed to imaging data in ophthalmology [1–4]. Deep learning (DL), a subset of AI, uses artificial neural networks to identify intricate patterns and structure in high-dimensional datasets [5]. These models are powerful tools for pattern recognition and classification. Proof-of-concept studies have shown that AI can potentially provide prognostic insights that can help select the optimal therapy and manage the expectations of patients and physicians [6, 7]. However, the production of these algorithms is limited to those with AI expertise and substantial financial resources.

Without coding knowledge, developing DL models is difficult for clinicians [1]. Fortunately, code-free deep learning (CFDL) offers a way for researchers and clinicians to produce highly accurate machine learning (ML) models without requiring any coding skills. It has been a major step in the democratisation of AI [8]. CFDL describes a set of tools and techniques for streamlining model development by automating the selection of optimal network architectures, pre-processing methods and hyperparameter optimization [9]. These models have been shown to rival hand-designed (bespoke) models [8]. However, these comparisons have limitations, as the models are developed by different teams and frequently utilize distinct datasets, which can affect the accuracy of the comparison [10]. Multiple studies explored the use of CFDL in the field of ophthalmology, including for electronic health record data and for traditional and ultra-widefield fundus photography [11–14]. The use of CFDL for medical videos is nevertheless limited. We published a study on cataract surgery phase classification using surgical videos where the CFDL model showed very good performance [15]. Another study looked at the use of CFDL for object detection from surgical videos in neurosurgery [16]. 

We designed a one-to-one comparative study evaluating the performance of multiclass CFDL models versus bespoke models at diagnosing retinal pathologies from OCT videos and images. These CFDL models were designed by an ophthalmology trainee without coding experience and were prospectively compared to models made by an AI expert. Both sets of models were created and assessed using the same dataset.

## Methods

### Study design

Using an internal OCT video dataset, we conducted a comparative analysis of the diagnostic capabilities of multiclass DL models developed through two distinct approaches. One model was created by an ophthalmology trainee without coding experience, leveraging CFDL technology, while another set was crafted by an experienced AI expert. These models had to classify OCT videos into five categories: normal/healthy retina, macular hole (MH), epiretinal membrane (ERM), wet age-related macular degeneration (AMD) and diabetic macular oedema (DME). Wet AMD was defined as neovascular AMD requiring anti-VEGF injection, regardless of the type of fluid, as assessed by an experienced retina specialist. We then designed multiclass models using fovea-centered images to diagnose those diseases using the same dataset. Incorporating both video and image data in our analysis allows for a robust comparison between the capabilities of CFDL technology and the skills of an AI expert, providing a solid foundation for evaluating their respective performances. Although no specific AI guidelines are available for this type of study, all efforts were made to report key terms and findings in adherence to CONSORT-AI extension [17]. 

Our ophthalmology trainee and AI expert carried out model development prospectively and simultaneously, but independently. All the models produced used supervised learning. The same dataset was used in both experiments and specific data splits (train and test) was randomly determined a priori allowing for fair comparison between the CFDL and bespoke models. The premise of this study was to demonstrate how CFDL can produce high-quality AI models without requiring a high level of human intervention and expertise. Since the ophthalmology trainee used a CFDL platform, they could not fine-tune the models to improve performance. However, we gave our AI expert as much time as needed to try out different model architectures to find the best one for our classification task.

### Data source

The videos used to train and test the models were prospectively collected at the Maisonneuve-Rosemont Hospital (Montreal, Quebec). The study was approved by the Institutional Review Board of the Maisonneuve-Rosemont Hospital (study #2021–2477) conforming to the principles of the Declaration of Helsinki. The macular scans were taken by experienced OCT technicians using a single device (Spectralis OCT machine (Heidelberg Engineering, Heidelberg, Germany)). Each scan consisted of a 25-line horizontal raster scan covering 20° × 20°, centered on the fovea. The videos were prospectively collected on a rolling basis among consecutive healthy patients and those with MH, ERM, AMD and DME who visited the retina service between December 2020 and December 2021. Image labeling was performed prospectively by an experienced vitreoretinal surgeon (RD) who had access to the patients’ charts and medical records. This labeling was deemed the ground truth and served as the benchmark against which the AI outputs were compared. No demographic or clinical data metadata was collected. The goal was to collect at least 100 scans from 100 patients per category over the study period, using only one scan per eye for each patient. To minimize data selection bias, all consecutive scans were collected regardless of age, gender, ethnicity or disease severity. No scans were excluded due to image quality during data curation. The uniformity in scan quality can be attributed to the specific patient population seen in the retina clinic. Patients referred to the clinic typically have a diagnostic OCT scan suggesting macular disease, prompting the referral to the retina service. As such, the scans in our dataset were likely of a higher quality.

We used OCT videos as they consist of a series of images captured during a single scanning session, showcasing a continuous, time-sequential view. This format closely mimics the clinical experience where a practitioner scrolls through an OCT scan in real-time. These video files were encoded in mp4 format and exported from a single device. They are not volume 3D scans, but rather videos showing sequential OCT B-scans over time. Each video was a sequence of frames with a frame rate of 10 frames per second. The duration of each video was 5 s, with each individual slice from the OCT scan being displayed across two consecutive frames. While this approach does not allow for volumetric analysis of fluid for example, we accepted this limitation since this was not the goal of this project. In fact, the main objective was to compare CFDL to bespoke models rather than producing models that are imminently deployable to clinic.

The total dataset contained 1,173 videos. From each video, a fovea-centered image (single OCT B-scan) was extracted to train and test the image model for a total of 1,173 images. Supplementary Fig. 1 shows representative images taken from the videos as well as fovea-centered images extracted for each category. Supplementary Table 1 shows the number of instances of each category and the distribution across training and testing. All classes had more than 100 training videos/images, with a median of 175 videos/images per class. Regarding the testing data, the median number of videos per phase was 19. Normal retina was the category with the highest number of instances and MH was the one with the lowest, reflecting local disease prevalence.

### CFDL model development

**Fig. 1:**
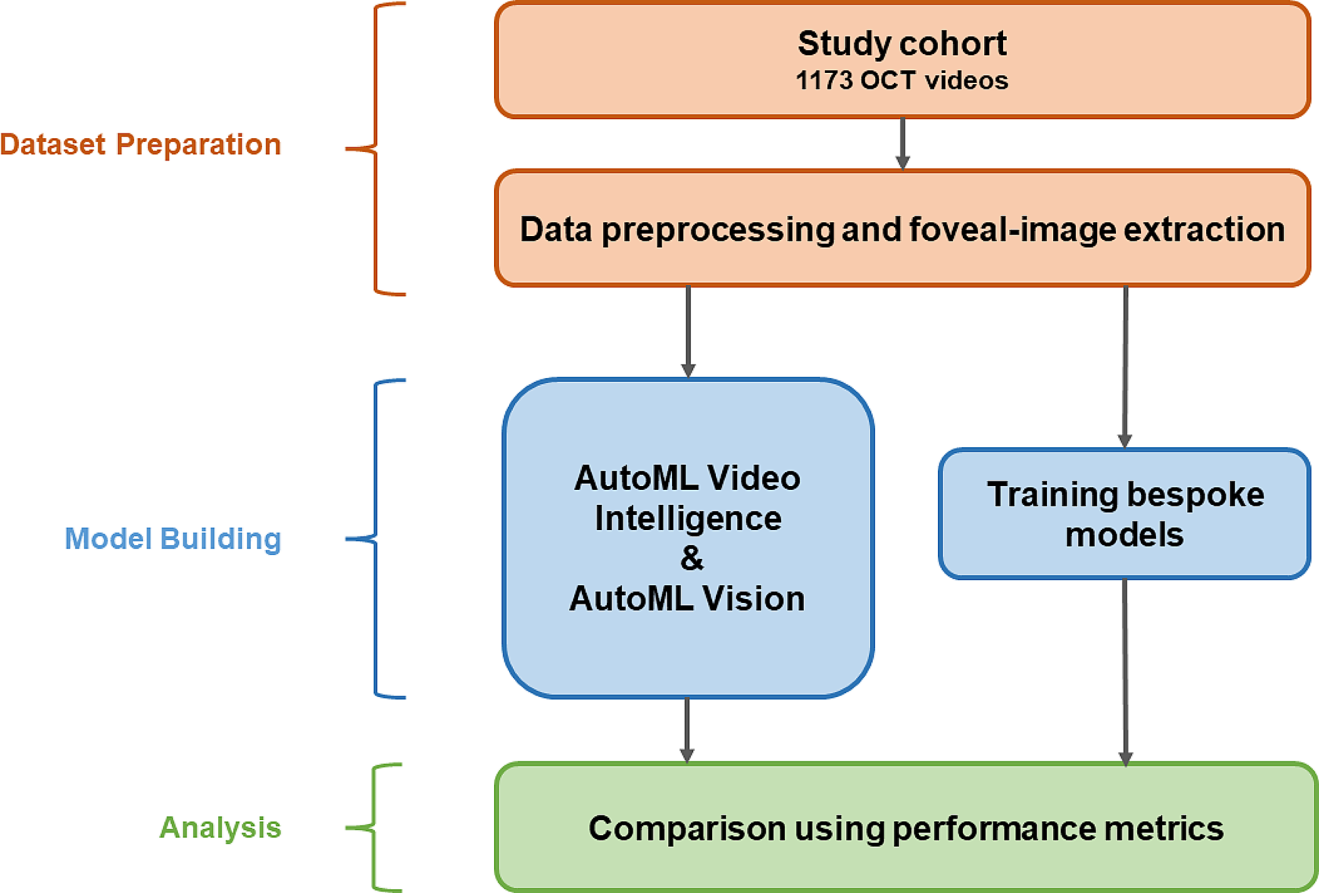
Overall workflow of the project

The overall workflow is presented in Fig. 1. An ophthalmology trainee without coding experience (ST) performed model development in Google Cloud AutoML Video Intelligence as well as in Google Cloud AutoML Vision. The videos and images were uploaded in a Google Cloud bucket with CSV files indicating the label/category, file path, and dataset distribution (i.e., training, or testing). We conducted each experiment only once, based on previous evidence of reasonable repeatability for AutoML training [9, 18]. We trained the cloud-hosted models for 17 node hours, as recommended by the platform, using our training data set size. To prevent overfitting, we used early stopping during the training process.

We split the data into two separate sets: 90% for training and validation, and 10% for testing. The platform then automatically divided the training group into training (70%) and validation (30%). Each video or image could only be used for either training or testing, but not both.

### Bespoke model development

The AI expert is a postdoctoral researcher with extensive expertise in DL research. The programing was carried out using Python V3.7. For the video classification task, we adapted the pre-trained CNN and Transformer architectures, including, Expand 3D networks (x3d-M, x3d-L) [27], r2plus1d-r50 [28], Multiscale Vision Transformers (mvit-base-32 × 3) [29], SlowFast Networks (slowfast-r50) [30], i3d-r50 [31], csn-r101 [32], ResNet3D (slow-r50) [33], TimeSformer-8 × 32-224 [34], and c2d-r50 [35]. All the models were fine-tuned to optimize categorical cross-entropy loss using the OCT video data set.

For the image classification task, we adopted a set of state-of the-art pre-trained convolutional neural networks and transformers, including Vision Transformer (ViT) [27], Swin Transformer (SwinT) [36], EfficientNetB0 [37], NasNetLarge [38], Xception [39], NasNetMobile [37]. Each model is fine-tuned using the OCT image data set. Cross-entropy is employed as a cost function to optimize the deep learning models.

All data processing and neural network training and prediction were accomplished in Python V.3.7 using PyTorch V.1.11.0, PyTorchVideo V.0.1.3, TensorFLow V2.7.0 and other related packages. The training was performed with a computer with AMD Ryzen 9 5950 × 16-Core (3.4 GHz) processor, 128 GB RAM, and GPU Nvidia GeForce RTX 3090 running on a 64-bit operating system.

The AI expert was initially blinded to the results of the CFDL models and carried out model development independently as they normally would for a classification task. Since the AutoML platform provided discriminative performance results within a couple of hours, we withheld the results from the AI expert for 30 days. The results were then disclosed and the AI expert was allowed any tools and techniques available to them, such as data augmentation, preprocessing, transfer learning, and hyperparameter optimization to match or surpass the CFDL models.

### Statistical analysis

The Google Cloud AutoML platform provides performance metrics that are commonly used and reported in the AI literature. These include area under the precision-recall curve (AUPRC), precision (positive predictive value, PPV) and recall (sensitivity). AUPRC is a metric ranging from 0 to 1.0 with higher values indicating better discriminative performance [17]. 

We also extracted data such as true positive (TP), true negative (TN), false positive (FP) and false negative (FN) with which we produced contingency tables. We also obtained the overall accuracy of the model by using the following formula: number of correctly classified images (or videos) divided by the total number of images (or videos) in the test set. These performance parameters were calculated to allow for a thorough comparison with the models developed by the expert.

Given that those models act as a diagnostic tool, opting for a threshold of 0.5 allows for a balanced trade-off between precision and recall. We compared the performance between the CFDL models and the bespoke ones via the use of four point-estimates: AUPRC, precision (PPV), sensitivity and accuracy.

### Saliency maps

To gain some understanding of the algorithms’ decision-making process, we produced saliency maps (also referred to as heatmaps) for the CFDL and bespoke image models. We used a region-based saliency method called XRAI (eXplanation with Ranked Area Integrals), which provides a high-level summary of insights. The viridis color map indicates which areas have the greatest influence or weight in the decision making. Yellow areas are those that influenced the most the prediction whereas those in blue played a lesser role [19]. These maps enable us to discern whether the CFDL models and custom-designed models rely on comparable image features when making their decisions.

## Results

Overall, the CFDL video model had excellent discriminative performance with an AUPRC of 0.984. At the 0.5 confidence threshold cut-off, the precision was 94.1%, the recall was 94.1% and the calculated accuracy for all categories was 94.1%. Table 1 displays the overall and per-category performance of the model. AUPRC ranged between 0.954 for ERM and 1.0 for both MH and wet AMD.

**Table 1:** Performance and evaluation of the CFDL video model

	Total	TP	FP	TN	FN	AUPRC	PPV	SN	ACC
**Overall**	118	NR	NR	NR	NR	0.984	94.1%	94.1%	94.1%
**Normal**	52	50	2	64	2	0.993	96.2%	96.2%	96.2%
**MH**	12	11	0	106	1	1.000	100.0%	91.7%	91.7%
**ERM**	21	19	3	94	2	0.954	86.4%	90.5%	90.5%
**AMD**	19	18	0	99	1	1.000	100.0%	94.7%	94.7%
**DME**	14	13	2	102	1	0.957	86.7%	92.9%	92.9%

TP, true positives; FP, false positives; TN, true negatives; FN, false negatives; AUPRC, area under the precision-recall curve; SN, sensitivity (recall); PPV, positive predictive value (precision); ACC, accuracy; MH, macular hole; ERM, epiretinal membrane; AMD, age-related macular degeneration; DME, diabetic macular oedema

Supplementary Table 2 presents the percentage of correct prediction and the most common misclassifications per class. The model accurately classified normal retina videos 96% of the time. Its lowest performance was with the ERM category, and still, the model was able to correctly identify 90% of the videos.

Table 2 displays the performance of the CFDL video model compared to the best model developed by the AI expert (MVit-base-32 × 3). We can see that for the AUPRC, the CFDL model had the highest value. Regarding accuracy, precision and sensitivity, the CFDL model came behind the Mvit-base-32 × 3 model with an extremely small difference (Google Cloud AutoML data’s precision stops at thousandths). Supplementary Table 3 shows the performance of the ten models produced by the AI expert.

**Table 2:** Performance of the CFDL video model compared to bespoke model

	Model	
	CFDL Video	Mvit-base-32 × 3
**AUPRC**	**0.984**	0.9097
**PPV**	0.941	**0.9419**
**Sensitivity**	0.941	**0.9419**
**Accuracy**	0.941	**0.9667**

CFDL, code-free deep learnin; AUPRC, area under the precision-recall curve; PPV, positive predictive value (precision)

The CFDL image model had an even better discriminative performance than the video model. The AUPRC was 0.990 and both precision and sensitivity were 96.6%. The overall accuracy of the algorithm was very high, reaching 95.8%. Supplementary Table 4 shows the detailed performance of the CFDL image model. AUPRC ranged between 0.975 for ERM and 1.0 for three categories (normal retina, MH and AMD).

**Fig. 2:**
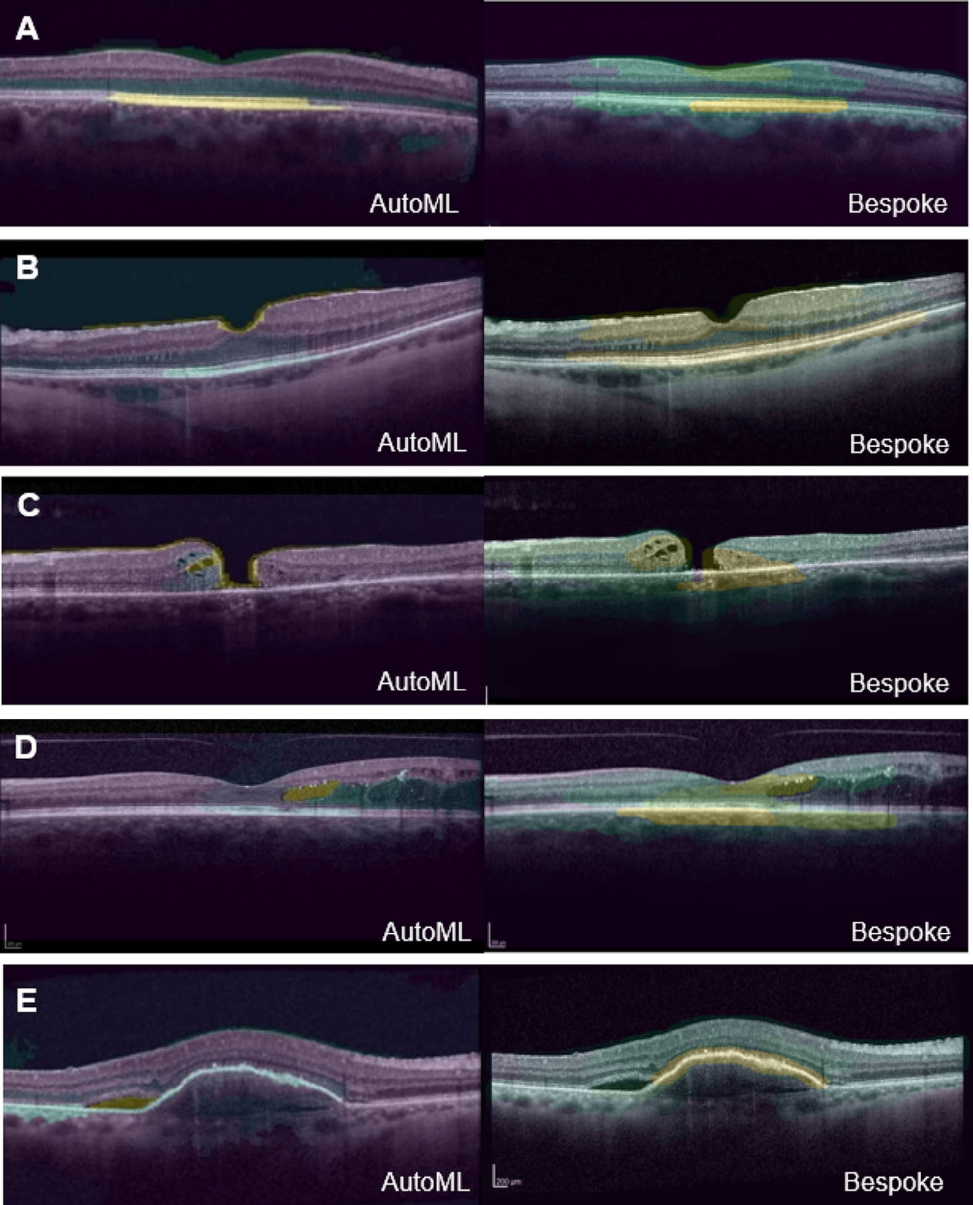
Saliency maps (XRAI method) of accurately predicted cases by the CFDL image model on the left and the best bespoke image (Xception) model on the right. Overall, both models highlighted similar areas as the most important region on OCT for the predicted class. (**A**) Normal macula: both saliency maps highlight the hyperreflective outer retinal layers, corresponding to the retinal pigmental epithelium/Bruch’s membrane complex and interdigitation zone. (**B**) Epiretinal membrane: the CFDL map highlights the epiretinal membrane/ internal limiting membrane while the bespoke map focuses on the overall foveal architecture. (**C**) Macular hole: both maps highlight the anvil-shaped deformity of the edges of the retina and the intraretinal edema. (**D**) Diabetic macular edema: both maps highlight intraretinal cystoid spaces. (**E**) Wet age-related macular degeneration: both maps highlight the elevation of the RPE. The CFDL map also highlights a small pocket of subretinal fluid

Table 3 presents the performance of the CFDL image model compared to the best bespoke algorithm (Xception). The CFDL image model had the same performance as the best bespoke model on all the metrics. Supplementary Table 5 displays the performance of all the image models produced by the AI expert. Figure 2 displays a saliency map for each of the five categories, showing the maps generated for the CFDL and bespoke image models. One correct prediction for each category was randomly selected to generate the saliency maps.

**Table 3:** Performance of the CFDL image model compared to bespoke model

	Model	
	CFDL Image	Xception
**AUPRC**	0.990	0.99
**PPV**	0.966	0.9658
**Sensitivity**	0.966	0.9658
**Accuracy**	0.966	0.9583

CFDL, code-free deep learning; AUPRC, area under the precision-recall curve; PPV, positive predictive value (precision)

## Discussion

In our comparative study, we evaluated the efficacy of code-free DL models against custom-designed models in accurately classifying retinal pathologies using OCT videos and images. This study provides a direct performance comparison between those two sets of models. The CFDL models were developed by an ophthalmology trainee with no coding experience and were prospectively compared to bespoke models produced by an AI expert. All the models in this study were produced on precisely the same dataset, with an identical distribution among the training and testing data. To our knowledge, the performance of CFDL video and image models has seldom been directly compared to state-of-the-art CNN and Transformer models in the same study. Previous CFDL research has often compared the performance of CFDL models to bespoke models developed by other teams, but it is difficult to ensure that the distribution of data between training and testing is the same in these cases. This is because open-access datasets often lack the necessary metadata indicating such splits [20]. Our study design allowed AI experts all tools at their disposition to match or surpass the CFDL model performance. Despite that, both researchers found very similar results– with CFDL requiring significantly less human involvement, time and resources.

Results from the CFDL models demonstrate robust overall performance for classifying retinal pathologies from OCT macular videos and images. For video classification, the AUPRC was 0.984 compared to 0.9097 for the pre-trained multiscale vision transformer model (Mvit-base-32 × 3 model). This result is impressive because transformers are considered remarkably successful in computer vision and video action recognition [21–24].. Similarly, the image classification model showed equivalent AUPRCs (0.99 and 0.99) between the CFDL model and the Xception model, a CNN. CFDL is able to produce such performing models through the use of three core techniques: transfer learning, neural architecture search and hyperparameter tuning [25]. Transfer learning is a technique where instead of building a model from scratch, you take advantage of pre-trained models that have been trained on similar datasets. Neural architecture search uses deep reinforcement learning to generate models that maximize performance on a given task [26]. 

We compared the performance between models trained on OCT macular videos and those trained solely on corresponding fovea-centered OCT images, in an attempt to determine the optimal data required for OCT model production. Interestingly, our results show that models trained on images outperformed those trained on videos, and so for both the CFDL and bespoke algorithms. We believe that the main reason for this difference is related to the ability to extract meaningful features from data, which seems to be done more effectively in images compared to videos. The videos might input a higher number of unnecessary information to the models, leading them to perform slightly less accurately. Of note, the performance of our image models could have been even better if the images were not centered on the fovea, but rather if we had selected the frame with the most evident pathological signs. However, we avoided this approach to prevent cherry-picking. Although our primary aim was not the development of an imminently deployable tool, our findings suggest that a potential model for retinal pathology diagnosis would likely achieve better performance with OCT images rather than videos.

We generated saliency maps to get insight into the model decision making process and ensure that the decisions were based on clinically relevant features (e.g., drusen, cystoid edema, etc.) rather than non-biological or spurious correlations. For the example labeled as normal, the models mainly looked at the presence of a continuous external limiting membrane (ELM) and retinal pigment epithelium (RPE). In the case of a wet AMD image, the model focused on the irregular form of the ELM/RPE and the presence of edema. Not only can these maps help us understand how the decisions are made, but they might even help us identify new or overlooked features on OCT to improve our own clinical judgment and interpretation of OCTs. This feature is not yet available in Google Cloud AutoML Video Intelligence. Using a similar technique, saliency maps were produced for the best bespoke model. These maps offer a different method for comparing code-free and handcrafted models by allowing us to observe whether similar features are being utilized by the different models. This approach has limitations but may provide a perspective on the underlying mechanisms and decision-making processes of each model type. The bespoke model’s maps highlight the same retinal layers and features as the one produced for the CFDL model, suggesting that the two models likely share a close architecture and framework.

Once the data is collected and labelled, producing algorithms using CFDL can be done in only a few hours. The user interacts with a simple drag-and-drop interface without writing any lines of code. This is an important advantage over handcrafted models that require extensive specialised expertise and computing resources. CFDL also appears to be more cost-effective than hiring an AI expert, which can make it more accessible for clinical research. Despite that, we do realise that CFDL is unlikely to fully replace the expertise and flexibility of AI engineers, but rather represents a tool that can make ML models accessible to clinicians and researchers with no programming experience. These experts have the ability to create and optimize models, provide a deeper understanding of the processes involved, and often possess knowledge in implementing the models on a large scale.

This study has some limitations. First, our testing was conducted on a single dataset. This raises the question of how the comparative performance of our models might fluctuate when applied to other datasets, especially those that differ in size and complexity. Despite this limitation, our comprehensive approach, encompassing both images and videos, allowed for a robust assessment of model performance. Second, it’s important to clarify that our study did not include an external validation. The decision to not perform one was aligned with our primary objective, which was to compare the capabilities of CFDL and bespoke AI technologies in a specific context, rather than to develop a clinically applicable tool. This focus on comparative analysis, rather than clinical application, guided our methodology. The internal dataset, composed of OCT videos and images, provided a sufficient basis for this comparison. Finally, as mentioned above, the OCT images were centered around the fovea rather than the main pathological findings, potentially lowering the performance of the model.

In summary, in this one-to-one comparative study, we demonstrated the feasibility for clinicians without coding experience to produce DL models via CFDL and also provided a detailed comparison with bespoke models crafted by an AI expert. Remarkably, the performance of the CFDL models paralleled that of the expert-designed ones, with both utilizing the same image features for decision-making. This finding underscores CFDL’s potential as a significant step towards democratizing AI in ophthalmology and broader medical fields. It opens up the possibility for researchers and clinicians without programming or data science expertise to create robust DL algorithms. Nevertheless, a strong collaboration between clinicians and AI experts is required to gain better understanding of the underlying processes and ultimately use these technologies to produce useful clinical applications.

### Electronic supplementary material

Below is the link to the electronic supplementary material.

Supplementary Material 1

Supplementary Material 2

## Data Availability

The dataset used and/or analysed during the current study are available from the corresponding author on reasonable request.
