# Supplementary material for: Comparing code-free deep learning models to expert-designed models for detecting retinal diseases from optical coherence tomography
Source: Int J Retina Vitreous. 2024 Apr 26;10:37. doi: 10.1186/s40942-024-00555-3 (PMC11055378; doi:10.1186/s40942-024-00555-3)
Supplement: Supplementary file 1 — Supplementary Material 1 [file 40942_2024_555_MOESM1_ESM.docx]

International Journal of Retina and Vitreous

Supplemental Material

**Comparing code-free machine learning models to expert-designed models for detecting retinal diseases from optical coherence tomography videos and photos**

Samir Touma^1,2,3^, Badr Ait Hammou^1,2^, Fares Antaki^1,2,3,4^, Marie Carole Boucher^1,2^, Renaud Duval^1,2^

1. Department of Ophthalmology, Université de Montréal, Montreal, Québec, Canada
2. Centre Universitaire d’Ophtalmologie (CUO), Hôpital Maisonneuve-Rosemont, CIUSSS de l’Est-de-l’Île-de-Montréal, Montreal, Quebec, Canada
3. Department of Ophthalmology, Centre Hospitalier de l'Université de Montréal (CHUM), Montreal, Quebec, Canada
4. The CHUM School of Artificial Intelligence in Healthcare (SAIH), Centre Hospitalier de l'Université de Montréal (CHUM), Montreal, Quebec, Canada

**Corresponding author:** Renaud Duval, MD, CM; Centre Universitaire d’Ophtalmologie (CUO), Hôpital Maisonneuve-Rosemont, CIUSSS de l’Est-de-l’Île-de-Montréal, 5415 boulevard de l'Assomption, Montréal, Québec, Canada, H1T 2M4. Email: [renaud.duval@gmail.com](mailto:renaud.duval@gmail.com)

| **Category** | **Training Data** | **Test Data** | **Total** | **Distribution** |
| --- | --- | --- | --- | --- |
| **Normal** | 469 | 52 | 521 | 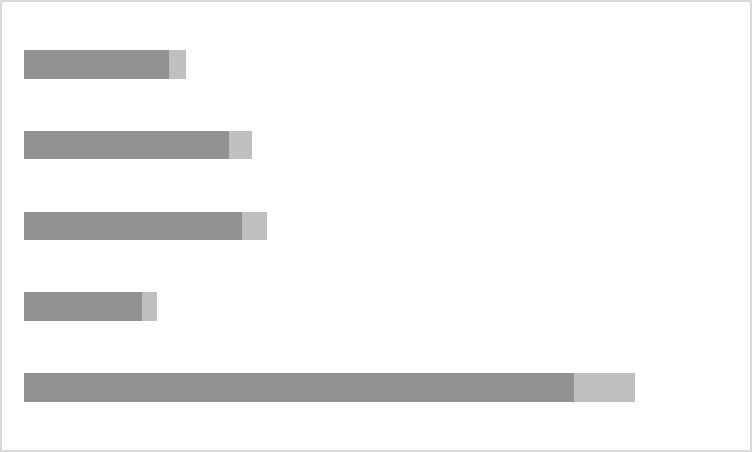 |
| **MH** | 101 | 12 | 113 |  |
| **ERM** | 186 | 21 | 207 |  |
| **Wet AMD** | 175 | 19 | 194 |  |
| **DME** | 124 | 14 | 138 |  |
| **Total** | **1055** | **118** | **1173** |  |
| MH, macular hole; ERM, epiretinal membrane; AMD, age-related macular degeneration; DME, diabetic macular oedema | | | | |

**Supplementary table 1. OCT videos and images distribution across training and testing.**

The dataset was split automatically by the automated machine learning (AutoML) platform: 90% for training (80% for actual training and 10% for validation), and 10% for testing. The median number of videos per category was 175 for the training data and 19 for the testing data.

| **True label** | **Correct prediction**  **N (%)** | **Misclassification** |  |
| --- | --- | --- | --- |
|  |  | **Most common**  **N (%)** | **2^nd^ most common**  **N (%)** |
| **Normal** | 50 (96%) | ERM : 2 (4%) | - |
| **MH** | 11 (92%) | ERM : 1 (8%) |  |
| **ERM** | 19 (90%) | Normal : 1 (5%) | DME : 1 (5%) |
| **Wet AMD** | 18 (95%) | DME : 1 (5%) | - |
| **DME** | 13 (93%) | Normal : 1 (7%) | - |
| MH, macular hole; ERM, epiretinal membrane; AMD, age-related macular degeneration; DME, diabetic macular oedema | | | |

**Supplementary table 2. Misclassification matrix for the AutoML video model**

The misclassification matrix presents the percentage of correct prediction and the most common misclassifications per category. The most accurately classified label was normal (96%), followed by wet AMD (95%). The model’s lowest performance was in the ERM category, being able to accurately identify 90% of the videos.

| **Model** | **AUPRC** | **PPV** | **SN** | **ACC** |
| --- | --- | --- | --- | --- |
| **AutoML Video Intelligence** | **0.984** | 0.941* | 0.941* | 0.941 |
| **Slowfast-r50** | 0.9096 | **0.9419** | **0.9419** | 0.9417* |
| **ResNet3D** | 0.9083 | 0.941* | 0.941* | 0.9083 |
| **C2d-r50** | 0.9036 | 0.9379 | 0.9379 | 0.925 |
| **I3d-r50** | 0.9037 | 0.9379 | 0.9379 | 0.9417* |
| **Csn-r101** | 0.9051 | 0.9388 | 0.9388 | 0.95 |
| **X3d-M** | 0.9046 | 0.9385 | 0.9385 | 0.925 |
| **R2plus1d-r50** | 0.9065 | 0.9398 | 0.9398 | **0.9667** |
| **Mvit-base-32x3** | 0.9097* | **0.9419** | **0.9419** | **0.9667** |
| **TimeSformer-8x32-224** | 0.8968 | 0.9333 | 0.9333 | 0.925 |
| **X3d-L** | 0.8981 | 0.9342 | 0.9342 | 0.9333 |
| AUPRC, area under the precision-recall curve; PPV, positive predictive value (precision); SN, sensitivity (recall); ACC, accuracy | | | | |

**Supplementary table 3. Performance of the AutoML video model compared to bespoke models**

Presents the performance of the AutoML model and the ten bespoke models. Per metric, the highest value is in bold and the second one has an asterisk. The AutoML model had the highest value for the AUPRC metric. The best bespoke model was the Mvit-base-32x3 with the best score in precision, sensitivity and accuracy.

|  | **Total** | **TP** | **FP** | **TN** | **FN** | **AUPRC** | **PPV** | **SN** | **ACC** |
| --- | --- | --- | --- | --- | --- | --- | --- | --- | --- |
| **Overall** | 118 | NR | NR | NR | NR | 0.990 | 96.6% | 96.6% | 95.8% |
| **Normal** | 52 | 50 | 0 | 65 | 2 | 1.000 | 100.0% | 96.2% | 96.2% |
| **MH** | 12 | 11 | 2 | 105 | 0 | 1.000 | 84.6% | 100.0% | 100.0% |
| **ERM** | 21 | 19 | 0 | 97 | 2 | 0.975 | 100.0% | 90.5% | 90.5% |
| **Wet AMD** | 19 | 19 | 1 | 98 | 0 | 1.000 | 95.0% | 100.0% | 100.0% |
| **DME** | 14 | 14 | 1 | 103 | 0 | 0.979 | 93.3% | 100.0% | 100.0% |
| MH, macular hole; ERM, epiretinal membrane; AMD, age-related macular degeneration; DME, diabetic macular oedema ,TP, true positive; FP, false positive; TN, true negative; FN, false negative; AUPRC, area under the precision-recall curve; PPV, positive predictive value (precision); SN, sensitivity (recall); ACC, accuracy | | | | | | | | | |

**Supplementary table 4. Performance and evaluation of the AutoML image model**

Presents the overall and per-category performance of the AutoML image. We can see that overall, the AUPRC was 0.99, precision 96.6%, recall 96.6% and accuracy 95.8%. The model had its highest performance when tested on wet AMD images, whereas it was the least accurate on ERM images.

| **Model** | **AUPRC** | **PPV** | **SN** | **ACC** |
| --- | --- | --- | --- | --- |
| **AutoML Vision** | **0.990** | **0.966** | **0.966** | **0.958** |
| **EfficientNet-B0** | 0.967 | 0.9417 | 0.9417* | 0.9417 |
| **NasNetLarge** | 0.9196 | 0.8974 | 0.875 | 0.9 |
| **Xception** | **0.99** | 0.9658*^∗^* | 0.9417* | **0.9583** |
| **NasNetMobile** | 0.8905 | 0.8269 | 0.7167 | 0.775 |
| **ViT** | 0.9797* | 0.9417 | 0.9417* | 0.9417 |
| **Swin-transformer (SwinT)** | 0.9776 | 0.9496 | 0.9417* | 0.95* |
| AUPRC, area under the precision-recall curve; PPV, positive predictive value (precision); SN, sensitivity (recall); ACC, accuracy | | | | |

**Supplementary table 5. Performance of the AutoML image model compared to bespoke models**

Presents the performance of the AutoML image models and the six bespoke models. Per metric, the highest value is in bold and the second one has an asterisk. The AutoML model had the highest value for the following metrics: AUPRC, PPV, SN and accuracy. The best bespoke model was the Xception.
