# Supplementary figures and images for: Comparing code-free deep learning models to expert-designed models for detecting retinal diseases from optical coherence tomography
Source: Int J Retina Vitreous. 2024 Apr 26;10:37. doi: 10.1186/s40942-024-00555-3 (PMC11055378; doi:10.1186/s40942-024-00555-3)

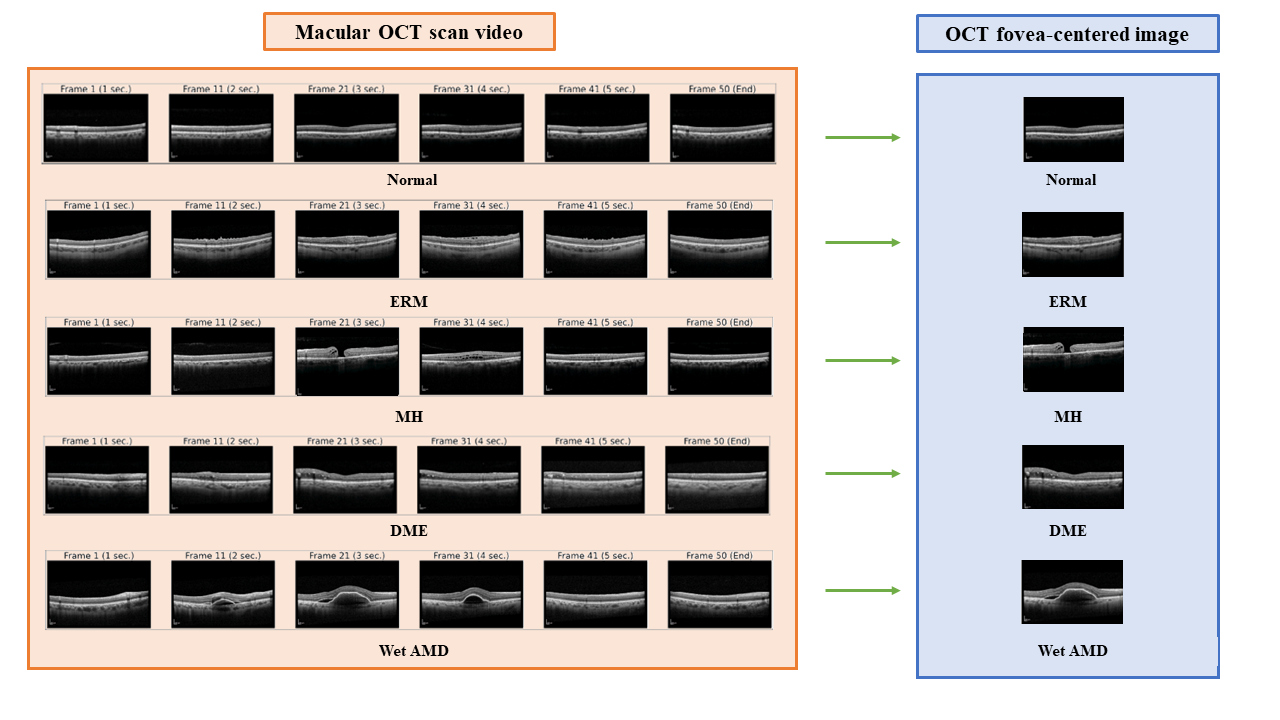

Supplement: Supplementary file 2 — Supplementary Material 2 [file 40942_2024_555_MOESM2_ESM.png]
